# SARS-CoV-2 Variants: Genetic Insights, Epidemiological Tracking, and Implications for Vaccine Strategies

**DOI:** 10.3390/ijms26031263

**Published:** 2025-01-31

**Authors:** Fatimah S. Alhamlan, Ahmed A. Al-Qahtani

**Affiliations:** 1 Department of Infection and Immunity, Research Centre, King Faisal Specialist Hospital & Research Centre, 11211 Riyadh, Saudi Arabia; falhamlan@kfshrc.edu.sa; 2 Department of Microbiology and Immunology, College of Medicine, Alfaisal University, 11211 Riyadh, Saudi Arabia

**Keywords:** SARS-CoV-2, Alpha, Delta, Omicron, epidemiological tracking, viral fitness, ACE-2 receptor

## Abstract

The emergence of SARS-CoV-2 variants has significantly impacted the global response to the COVID-19 pandemic. This review examines the genetic diversity of SARS-CoV-2 variants, their roles in epidemiological tracking, and their influence on viral fitness. Variants of concern (VOCs) such as Alpha, Beta, Gamma, Delta, and Omicron have demonstrated increased transmissibility, altered pathogenicity, and potential resistance to neutralizing antibodies. Epidemiological tracking of these variants is crucial for understanding their spread, informing public health interventions, and guiding vaccine development. The review also explores how specific mutations in the spike protein and other genomic regions contribute to viral fitness, affecting replication efficiency, immune escape, and transmission dynamics. By integrating genomic surveillance data with epidemiological and clinical findings, this review provides a comprehensive overview of the ongoing evolution of SARS-CoV-2 and its implications for public health strategies and new vaccine development.

## 1. Introduction

Severe Acute Respiratory Syndrome Coronavirus 2 (SARS-CoV-2), also known as the novel coronavirus, is a member of the coronaviridae family [1]. It was first identified in December 2019 in Wuhan, China, and has since caused a global pandemic known as COVID-19. SARS-CoV-2 is an enveloped virus with a spherical shape [2]. The virus has spike (S) proteins on its surface, which give it a crown-like appearance under an electron microscope, hence the name “corona” (Latin for crown). These spike proteins play a crucial role in the virus’s ability to infect host cells by binding to the angiotensin-converting enzyme 2 (ACE2) receptor in human cells [3]. The genome of SARS-CoV-2 is a single-stranded positive-sense RNA molecule approximately 29.9 kilobases in length [2]. The genome encodes for several structural proteins, including the spike (S), envelope (E), membrane (M), and nucleocapsid (N) proteins, as well as several nonstructural proteins that are involved in the virus’s replication and transcription processes [1]. The RNA genome is organized into a 5′ cap, a leader sequence, and a body that contains open reading frames (ORFs) encoding for the viral proteins, followed by a 3′ untranslated region (UTR) and a poly (A) tail [1].

The initial crucial phase of viral infection is driven by the trimeric spike protein, which is present on the virus’s surface as a key antigen and triggers neutralizing antibody responses [4]. Consequently, this protein is a vital focus for the creation of diagnostics, treatments, and vaccines. Since the virus first emerged, significant advancements have been achieved in understanding the structural biology of the SARS-CoV-2 S protein. This review provides a comprehensive overview of the structural and genetic characteristics of SARS-CoV-2 variants, highlighting their critical role in epidemiological tracking. It addresses the existing knowledge gaps by examining how these variants impact the virus’s fitness for survival and its evolutionary trajectory.

## 2. SARS-CoV-2 Variants

SARS-CoV-2, the causative agent of COVID-19, has undergone significant genetic mutations since its emergence in late 2019. These mutations have led to the emergence of various variants, each with distinct characteristics and implications for public health. This review aims to provide a comprehensive overview of SARS-CoV-2 variants, drawing on multiple published sources.

### 2.1. Genetic Variability and Evolution

SARS-CoV-2 variants are characterized by mutations in the viral genome, particularly in the spike (S) protein, which plays a crucial role in the virus’s ability to infect host cells (Table 1). The spike protein is the primary target for neutralizing antibodies and vaccines, making mutations in this region particularly significant. Variants such as Alpha (B.1.1.7), Beta (B.1.351), Gamma (P.1), Delta (B.1.617.2), and Omicron (B.1.1.529) have been identified and classified by the World Health Organization (WHO) as variants of concern due to their increased transmissibility, virulence, or resistance to neutralizing antibodies.

#### 2.1.1. Alpha Variant (B.1.1.7) of SARS-CoV-2

The Alpha variant (B.1.1.7) was first identified in the United Kingdom in September 2020. It was reported to the WHO in December 2020 and quickly became a variant of concern due to its rapid spread and significant mutations [5]. The Alpha variant demonstrated a substantial increase in transmissibility compared to previous strains. It rapidly spread across the UK and other countries, becoming the dominant strain in many regions by early 2021. Studies estimated that the Alpha variant was 50–100% more transmissible than earlier variants [5].

The Alpha variant is characterized by 23 mutations, including 17 non-synonymous mutations, 6 synonymous mutations, and 3 deletions [6]. Key mutations in the spike protein include N501Y, which increases binding affinity to the ACE2 receptor, and the deletion of amino acids 69–70 (Δ69–70), which is associated with immune evasion and increased infectivity [6,7].

##### Impact of Mutations on Virulence and Morbidity

**Figure 1:**
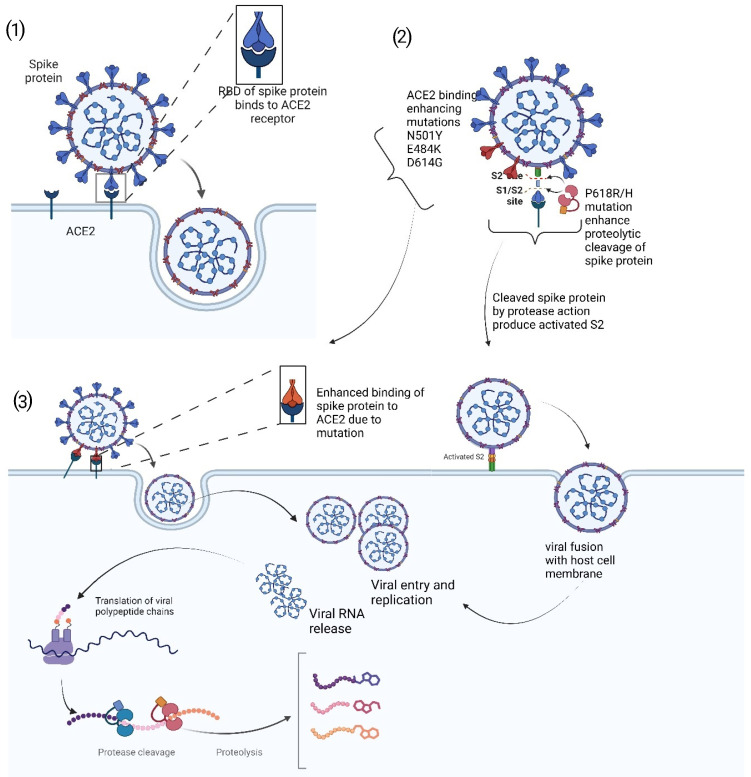
SARS-CoV-2 mutations and viral fitness: The binding of SARS-CoV-2 spike protein to the ACE2 receptor facilitates the entry of the virus into a host cell (**1**), but this entry is enhanced by specific mutations such as N501Y, E484K, and D614G (**2**). These mutations enhance spike protein affinity for the ACE2 receptor (**3**), thus increasing the number of viruses binding to host cells and, subsequently, increasing viral load in host cells. Similarly, P618R mutation near the furin cleavage site facilitates the proteolytic cleavage of the spike protein at the S1/S2 site (**2**) activating the S2, which also increases the fusion of the virus to host membrane (**3**). This also invariably increases the viral entry into the host cell.

Epidemiological estimates in the United Kingdom showed that the Alpha variant may be more transmissible than the original SARS-CoV-2 lineage, becoming the dominant strain in the United Kingdom by the first quarter of 2021 [8]. The mutations in the Alpha variant have been linked to increased virulence. The N501Y mutation enhances the ability of the virus to bind to the ACE2 receptor, facilitating easier entry into human cells (Figure 1). This mutation, along with others, contributes to the variant’s higher transmissibility and, potentially, increased disease severity [9].

In addition to N501Y, the Alpha variant includes other mutations, such as the deletion of amino acids 69–70 (Δ69–70) and P681H. The Δ69–70 deletion is associated with immune evasion, allowing the virus to partially escape detection by the immune system [10,11]. This deletion, along with the N501Y mutation, contributes to the variant’s increased transmissibility. Furthermore, the P681H mutation is located near the furin cleavage site of the spike protein. This has been thought to not necessarily enhance the virus’s ability to enter cells by facilitating the cleavage of the spike protein, a critical step for viral entry, but may contribute to the increased transmissibility and, potentially, higher virulence of the Alpha variant [12].

Subsequent to the high transmissibility and virulence of the Alpha variant, the strain has been associated with higher morbidity rates. Studies have shown that infections with the Alpha variant may be responsible for increased hospitalization rates and a higher risk of severe disease outcomes [13,14]. The variant’s enhanced transmissibility and potential for more severe disease have placed significant strain on healthcare systems.

#### 2.1.2. Beta Variant (B.1.351) of SARS-CoV-2

The Beta variant, also known as B.1.351, was first identified in South Africa in May 2020 and was reported to the WHO in December 2020. It became a variant of concern due to its mutations that impacted transmissibility and immune evasion [10]. The Beta variant demonstrated increased transmissibility compared to earlier strains, contributing to regional surges in cases [15]. It also exhibited a significant ability to evade immunity, posing challenges to vaccine effectiveness and increasing the risk of reinfection.

The Beta variant is characterized by several key mutations in the spike protein, including K417N, E484K, and N501Y. The E484K mutation is strongly associated with immune escape, enabling the virus to evade neutralizing antibodies generated by prior infection or vaccination. The N501Y mutation enhances the binding affinity to the ACE2 receptor, increasing infectivity. Additionally, K417N contributes to immune evasion and reduced neutralization by monoclonal antibodies [16].

##### Impact of Mutations on Virulence and Morbidity

The mutations in the Beta variant have been associated with reduced efficacy of neutralizing antibodies, making it more challenging to control infections [17]. The immune escape properties of E484K and K417N mutations have been linked to higher rates of reinfection and breakthrough cases among vaccinated individuals [18]. While the Beta variant has not demonstrated significantly increased virulence compared to other variants, its immune evasion capabilities have led to extended periods of transmission, particularly in regions with limited vaccine coverage [19].

##### Beta Variant and Its Impact on Mortality

Although the Beta variant has been associated with increased transmissibility and immune evasion, its impact on mortality has been less pronounced compared to Delta. Vaccines retained partial effectiveness in preventing severe disease and death caused by the Beta variant, particularly with mRNA-based vaccines [20]. However, in populations with low vaccine coverage, the Beta variant contributed to increased hospitalizations and mortality rates during its peak periods of circulation [21].

#### 2.1.3. Gamma Variant (P.1) of SARS-CoV-2

The Gamma variant, also known as P.1, was first detected in Manaus, Brazil, in November 2020 and reported to the WHO in January 2021. It became a variant of concern due to its rapid spread in South America and its potential for immune evasion [22]. The Gamma variant demonstrated increased transmissibility and was implicated in a resurgence of cases in regions that had previously experienced high infection rates, suggesting a heightened risk of reinfection [23].

The Gamma variant is characterized by key mutations in the spike protein, including K417T, E484K, and N501Y. These mutations enhance binding to the ACE2 receptor (N501Y) and confer immune evasion properties (E484K and K417T). The combination of these mutations has been associated with higher infectivity and partial resistance to neutralizing antibodies [24].

##### Impact of Mutations on Virulence and Morbidity

The Gamma variant’s mutations contributed to higher viral loads and a moderate increase in disease severity. Studies found that individuals infected with Gamma had a higher risk of severe disease compared to those infected with earlier variants, though not as pronounced as the Delta variant [25]. Immune evasion properties of E484K and K417T mutations led to reinfections and reduced vaccine effectiveness, particularly for non-mRNA vaccines [26].

##### Gamma Variant and Its Impact on Mortality

The Gamma variant was associated with increased mortality in regions with limited healthcare resources and low vaccination rates. The surge in cases overwhelmed healthcare systems in South America, contributing to higher mortality rates during periods of Gamma dominance [27]. However, in populations with widespread vaccination, the impact of Gamma on mortality was mitigated, with vaccinated individuals experiencing less severe disease and lower mortality rates [28].

#### 2.1.4. Delta Variant (B.1.617.2) of SARS-CoV-2

The Delta variant, also known as B.1.617.2, was first identified in India in October 2020. It was reported to the WHO in May 2021 and quickly became a variant of concern due to its rapid spread and significant mutations [29]. The Delta variant demonstrated a substantial increase in transmissibility compared to the Alpha strain. It rapidly spread across India and other countries, becoming the dominant strain globally by mid-2021, thus becoming a variant of concern. Studies estimated that the Delta variant was up to 60% more transmissible than the Alpha variant, which itself was already highly transmissible [30,31]. The Delta variant is characterized by several key mutations in the spike protein, including L452R, T478K, and P681R. The L452R mutation enhances the virus’s ability to bind to the ACE2 receptor, increasing infectivity. The T478K mutation is associated with immune escape, allowing the virus to partially evade neutralizing antibodies. The P681R mutation, located near the furin cleavage site, facilitates more efficient viral entry into host cells [32].

##### Impact of Mutations on Virulence and Morbidity

The mutations in the Delta variant have been linked to increased virulence. The L452R and P681R mutations are known to enhance cleavage of this spike protein, thus enhancing the virus fusogenicity and its ability to infect cells [33]. This subsequently facilitates the spread within the host and the development of higher viral loads. This increased viral load is associated with more severe disease outcomes [34]. Additionally, both L452R and T478K mutations contribute to immune evasion, reducing the effectiveness of neutralizing antibodies that are secreted during infection and, potentially, leading to higher rates of reinfection and breakthrough infections in vaccinated individuals [35].

The Delta variant has been associated with higher morbidity rates. Studies have shown that infections with the Delta variant lead to increased hospitalization rates and a higher risk of severe disease outcomes. Studies have indicated that individuals infected with the Delta variant are more likely to require hospital care compared to those infected with previous variants. For instance, a previous study found that the risk of attending a hospital for emergency care or being admitted to a hospital within 14 days of infection with the Delta variant was 1.45 times greater compared to the Alpha variant [36]. This increased risk was particularly pronounced among unvaccinated individuals, who constituted the majority of hospital admissions during the Delta wave [36].

The increased transmissibility and severity of the Delta variant have placed significant strain on healthcare systems worldwide [37]. During periods of Delta variant dominance, hospitals experienced higher numbers of COVID-19 patients, leading to overcrowded emergency departments and ICU units. The increased demand for medical care also strained healthcare resources, including staffing, medical supplies, and hospital beds [38].

##### Delta Variant and Its Impact on Mortality

While the Delta variant has been linked to higher morbidity, its impact on mortality rates has been complex. Some studies suggest that the Delta variant may lead to higher mortality rates, particularly among unvaccinated populations [39]. However, the widespread rollout of vaccines has mitigated some of these effects. Vaccinated individuals, even if infected with the Delta variant, generally experience less severe disease and lower mortality rates compared to unvaccinated individuals [39].

#### 2.1.5. Omicron Variant (B.1.1.529) of SARS-CoV-2

The Omicron variant, also known as B.1.1.529, was first identified in South Africa in November 2021. It was reported to the WHO shortly thereafter and was quickly classified as a variant of concern due to its significant number of mutations and rapid spread [40]. The Omicron variant demonstrated a substantial increase in transmissibility compared to previous strains. It rapidly spread across the globe, leading to significant waves of infection. Early studies indicated that Omicron was more transmissible than the Delta variant, with a basic reproduction number (R0) estimated to be significantly higher [41,42]. The R0, which indicates the average number of people to whom a single infected person will transmit the virus, is a key measure of transmissibility. For the Delta variant, the R0 was estimated to be between 5 and 8, indicating a high level of transmissibility [42]. However, the Omicron variant’s R0 was found to be even higher. Danish researchers estimated that the effective reproduction number of Omicron is approximately 3.19 times greater than that of Delta [43]. Similarly, another comparative analysis of all the main variants showed that Omicron is 4.2 times more transmissible than Delta early in its spread [44].

##### Mutations and Subvariants

The Omicron variant is characterized by over 30 mutations in the spike protein, including several in the receptor-binding domain (RBD). Its primary subvariants—BA.1, BA.2, BA.3, BA.4, and BA.5—exhibit distinct mutation profiles, influencing their spread and immune escape potential. BA.1 was the first major Omicron subvariant to emerge, driving the initial global wave of Omicron infections [19]. It included key mutations such as G339D, S371L, and N501Y, which enhanced its transmissibility and ability to evade immunity [29]. BA.2, known as the “stealth variant” due to its lack of the 69–70 deletion used to differentiate BA.1 from Delta in PCR tests, demonstrated even higher transmissibility than BA.1 and rapidly became the dominant strain in many regions [45]. BA.3, on the other hand, shared mutations with both BA.1 and BA.2 but had lower fitness, limiting its global impact. Subsequently, BA.4 and BA.5 emerged with mutations such as L452R and F486V, which further enhanced immune evasion and receptor binding, making BA.5 the globally dominant subvariant by mid-2022 [46].

##### Recombinant XBB Subvariants

Recombinant variants, such as XBB and its descendants, arose when two distinct SARS-CoV-2 lineages infected the same host and exchanged genetic material [47]. XBB was formed from BA.2.10.1 and BA.2.75, exhibiting enhanced immune evasion but with severity comparable to earlier Omicron subvariants [48]. Among its descendants, XBB.1.5, often referred to as “Kraken”, garnered global attention due to its high transmissibility and partial resistance to neutralizing antibodies, driven by mutations like F486P [49]. Another notable subvariant, XBB.1.16, or “Arcturus”, gained prominence for its role in rising case numbers in specific regions, attributed to its superior immune escape capabilities [50]. While these recombinant subvariants have not demonstrated increased severity compared to other Omicron subvariants, their potential for rapid spread and immune evasion remains a significant concern.

##### Impact of Mutations on Virulence and Morbidity

The mutations in the Omicron variant have been linked to changes in virulence [51]. While Omicron is associated with increased transmissibility, initial studies suggested that it might cause less severe disease compared to the Delta variant [51,52]. This could be due to several factors, including the variant’s altered tropism, which may favor the upper respiratory tract over the lower respiratory tract, potentially leading to less severe respiratory symptoms [53]. Other suggestions are that the increased vaccination rate at the time of spread may have reduced the morbidity of the variant [15,51].

Despite its potentially lower virulence, the Omicron variant has led to significant morbidity due to its high transmissibility [51]. The large number of infections resulted in increased hospitalizations, particularly among unvaccinated individuals and those with underlying health conditions [54]. However, studies have shown that the risk of severe outcomes, including hospitalization and death, is lower for Omicron compared to Delta, especially among vaccinated populations [55,56].

#### 2.1.6. Non-VOC Variants of SARS-CoV-2

**Figure 2:**
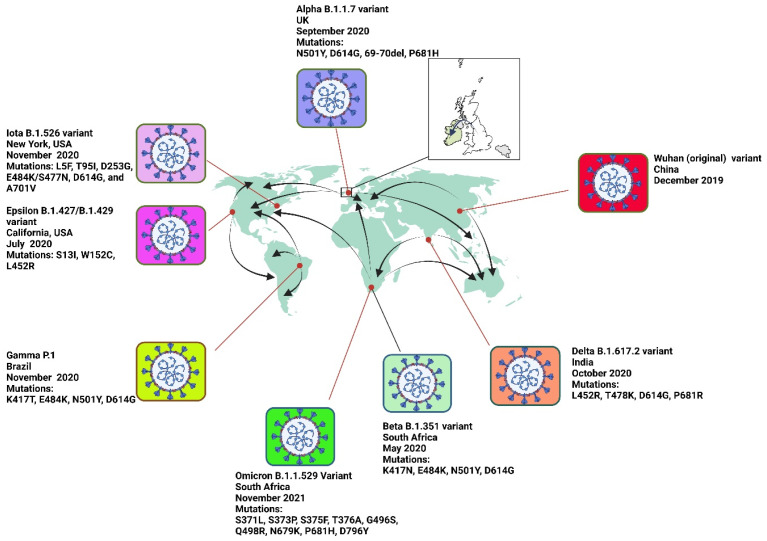
The map highlights the following variants: Alpha (B.1.1.7), which originated in the United Kingdom in September 2020 and spread to the United States, Canada, Germany, France, and Japan; Beta (B.1.351), which originated in South Africa in May 2020 and spread to the United States, United Kingdom, France, Australia, and South Korea; Gamma (P.1), which originated in Brazil in November 2020 and spread to Japan, the United States, Canada, Italy, and Argentina; Delta (B.1.617.2), which originated in India in October 2020 and spread to over 100 countries, including the United States, United Kingdom, Russia, Australia, and South Africa; Omicron (B.1.1.529), first detected in South Africa in November 2021 and spread rapidly to nearly every country, including the United States, United Kingdom, Germany, France, and Japan; BA.2.86 (Nextstrain clade 23I), detected in July 2023 in countries such as the United States, United Kingdom, Denmark, Israel, and South Africa; JN.1 (Nextstrain clade 24A), detected in August 2023 and spread to the United States, United Kingdom, India, Brazil, and Australia; Epsilon (B.1.427/B.1.429), which originated in California, USA, in July 2020 and spread to Mexico, Canada, South Korea, Japan, Australia, Germany, France, and the United Kingdom; and the Wuhan (original) variant, which originated in Wuhan, China, in December 2019 and spread globally, reaching countries such as Italy, Iran, South Korea, and the United States by early 2020. Arrows indicate the spread of each variant from its origin to other regions.

In addition to VOCs, several Variants of Interest (VOIs) were identified during the pandemic, including Epsilon (B.1.427/B.1.429) and Iota (B.1.526) (Figure 2). While these variants initially raised concerns due to their mutations linked to increased transmissibility and immune escape, their impact remained localized or limited. Over time, these variants were outcompeted by more transmissible and immune-evasive VOCs like Delta and Omicron, leading to their downgrading from VOI status.

##### Epsilon Variant (B.1.427/B.1.429)

The Epsilon variant, first identified in California, USA, in May 2020, was designated a Variant of Interest (VOI) by the WHO in March 2021 but later downgraded due to reduced global impact [57]. Epsilon is characterized by key spike protein mutations L452R and S13I, which enhanced transmissibility and partial immune escape [58]. It showed moderate resistance to neutralizing antibodies from prior infections and vaccines. However, its spread remained geographically limited, and it was eventually outcompeted by more transmissible variants like Delta and Omicron.

##### Iota Variant (B.1.526)

The Iota variant, first detected in New York, USA, in November 2020, was designated a VOI in March 2021 [59]. It featured mutations such as E484K and D614G, associated with immune escape and increased infectivity [60]. Early studies suggested that Iota could evade some neutralizing antibodies, but its impact was overshadowed by the emergence of more transmissible variants [61]. Like Epsilon, Iota was later downgraded due to its declining prevalence and reduced public health threat globally.

## 3. Role of SARS-CoV-2 Variants in Viral Fitness

Viral fitness refers to the ability of a virus to successfully replicate and transmit within a host population. For SARS-CoV-2, the emergence of various variants has significantly impacted its fitness, influencing factors such as transmissibility, infectivity, and immune evasion. This section explores how specific mutations in SARS-CoV-2 variants enhance viral fitness and the implications for public health.

### 3.1. Increased ACE2 Receptor Affinity

One of the primary ways SARS-CoV-2 variants enhance viral fitness is through mutations that increase the virus’s affinity for the ACE2 receptor, which is the entry point for the virus into human cells. The Alpha variant (B.1.1.7) contains the N501Y mutation in the spike protein, which enhances binding to the ACE2 receptor, facilitating easier entry into host cells and increasing infectivity [6,7]. This ACE2 affinity mutation in the spike protein was detected early during the pandemic in different strains and has since remained in the variants likely due to its importance in ACE2 binding for enhancing transmissibility [62]. Similarly, the Delta variant (B.1.617.2) has the L452R mutation, which also improves ACE2 binding and contributes to higher transmissibility and even resistance to vaccines. In fact, some studies have demonstrated that the L452R mutation confers the virus with resistance to mRNA-based vaccines from different companies [4,63,64].

### 3.2. Enhanced Transmissibility

Mutations that increase the transmissibility of the virus are another key factor in viral fitness. The Delta variant, for instance, demonstrated a significant increase in transmissibility compared to earlier strains, partly due to the P681R mutation near the furin cleavage site, which enhances the virus’s ability to enter cells (See Section 2.1.2) [32]. Similarly, the Omicron variant (B.1.1.529) also showed increased transmissibility, with multiple mutations in the spike protein contributing to its rapid spread. These mutations allow the virus to spread more efficiently within populations, leading to larger outbreaks and higher case numbers.

### 3.3. Immune Evasion

Variants that can evade the immune response have a fitness advantage as they can infect individuals who have been previously infected or vaccinated. A primary concern during the early evolution of SARS-CoV-2 was the potential development of antigenically distinct variants capable of evading immunity acquired through vaccination or previous infection, such as the N439K spike mutation [65]. Before the COVID-19 vaccine update in late 2022, all widely available vaccines were developed based on the spike antigen of early variants, which were mostly from the reference sequence Wuhan-Hu-1, which often harbors mutations stabilizing the spike protein in a prefusion conformation [66]. Although the Alpha variant exhibited minimal antigenic changes, laboratory experiments revealed that the Beta, Gamma, and Delta variants demonstrated moderate immune escape from vaccine-induced antibodies and convalescent sera [67,68,69].

The Beta variant (B.1.351) and the Omicron variant both have mutations such as E484K, termed the escape mutation, and E484A, respectively, which reduce the effectiveness of neutralizing antibodies [70]. The Omicron ’complex’, which includes the distinct sublineages BA.1 to BA.5, can infect both vaccinated individuals and those previously infected, highlighting the urgent need for updated vaccine sequences and universal vaccines in SARS-CoV-2 control strategies [19]. These sublineages possess over 15 mutations in the spike RBD and several antigenic deletions and substitutions in the amino-terminal domain (NTD). Consequently, BA.1, BA.2, BA.4, and BA.5 are poorly neutralized by first-generation vaccines and antibodies derived from pre-Omicron infections [68,71,72]. Additionally, these sublineages have shown resistance to most current therapeutic monoclonal antibodies, with bebtelovimab, a monoclonal antibody targeting the RBD of the spike protein, being one of the few that retains efficacy against all SARS-CoV-2 variants [19].

### 3.4. Increased Viral Replication

Some mutations enhance the virus’s ability to replicate within the host, contributing to higher viral loads and increased transmission. For example, the Delta variant’s combination of mutations, including L452R and P681R, not only improves ACE2 binding but also enhances viral replication [73]. Structural analyses have shown that the L452R mutation causes conformational changes that stabilize the interaction between the spike protein and the ACE2 receptor, thereby increasing infectivity [74]. In vitro studies have demonstrated that pseudoviruses carrying the L452R mutation exhibit significantly higher entry efficiency into host cells, with a 6.7–22.5-fold increase in 293T cells and a 5.8–14.7-fold increase in human airway lung organoids compared to the D614G variant (Figure 1) [32].

The P681R mutation enhances the cleavage of the spike protein into its S1 and S2 subunits, a necessary step for the virus to fuse with the host cell membrane and initiate infection (Figure 1). A previous study showed that the P681R mutation significantly increased the efficiency of this cleavage process, leading to more effective viral entry and higher replication rates [75]. It was demonstrated that the Delta variant with the P681R mutation outcompeted the Alpha variant in a competition assay, demonstrating superior replication fitness [75]. Invariably, higher viral loads can lead to more severe disease and greater potential for transmission, further increasing the variant’s fitness.

## 4. Variant Verification and Its Role in Epidemiological Tracking

Variant verification is a critical component in the ongoing battle against COVID-19. As SARS-CoV-2 continues to evolve, the identification and verification of new variants are essential for understanding the virus’s behavior, guiding public health responses, and informing vaccine development. This section explores the importance of variant verification and its role in epidemiological tracking.

### 4.1. Importance of Variant Verification

Variant verification is essential for tracking the genetic changes in SARS-CoV-2, enabling scientists to understand how the virus evolves and adapts. By identifying specific mutations, researchers can gain insights into the mechanisms driving viral evolution. This knowledge is crucial for predicting future changes and preparing for potential new variants.

SARS-CoV-2, like other RNA viruses, exhibits high genetic variability, particularly in the spike protein region [76]. This variability is driven by mutations that can enhance the virus’s ability to infect hosts, evade immune responses, and increase transmissibility. For instance, the D614G mutation, which became dominant early in the pandemic as alluded to earlier, was associated with increased viral fitness and transmissibility [77]. Similarly, continuous genomic surveillance and sequencing are vital for identifying and tracking new mutations. For example, the emergence of the Alpha variant (B.1.1.7) was marked by several key mutations, including N501Y, which increased the virus’s binding affinity to the ACE2 receptor, facilitating easier entry into human cell infectivity [6,7]. Furthermore, the Delta variant (B.1.617.2) exhibited mutations such as L452R and P681R, which enhanced its transmissibility and virulence [32]. As such, continuous genomic surveillance and sequencing enables the timely identification and tracking of new mutations, which is essential for understanding and mitigating the impacts of more infectious and virulent SARS-CoV-2 variants as well as the pattern of spread globally (Figure 2).

By understanding the evolutionary trajectory of SARS-CoV-2, researchers can predict potential future variants. This involves analyzing the selective pressures acting on the virus, such as immune responses and antiviral treatments. For instance, the Omicron variant (B.1.1.529) emerged with over 30 mutations in the spike protein, many of which were associated with immune escape and increased transmissibility [78]. Such insights are critical for updating vaccines and therapeutic strategies. The ability to rapidly identify and verify new variants allows public health officials to implement timely interventions. For example, the rapid spread of the Delta variant prompted increased vaccination efforts and the reintroduction of certain public health measures to curb transmission [79]. Understanding the genetic changes in SARS-CoV-2 also helps in assessing the effectiveness of existing vaccines and the need for booster doses [80]. According to a study, the Delta variant’s higher transmissibility and potential for increased severity of disease necessitated enhanced vaccination campaigns and booster doses to maintain immunity levels [81].

In response to the Delta variant, many countries accelerated their vaccination programs. For example, the United States saw a significant push to increase vaccination rates, particularly in areas with low coverage [82]. This included expanding eligibility for vaccines, setting up mass vaccination sites, and implementing mobile vaccination units to reach underserved communities [82]. Additionally, booster doses were recommended to enhance protection against the Delta variant, especially for vulnerable populations and those with waning immunity [83].

Public health measures such as mask mandates, social distancing, and travel restrictions were also reintroduced in many regions to control the spread of the Delta variant. A study in *Lancet* highlighted that these measures, combined with vaccination, were effective in reducing transmission rates and preventing healthcare systems from becoming overwhelmed [84]. The reintroduction of these measures was based on the understanding that the Delta variant’s mutations, such as L452R and P681R, increased its ability to spread and evade immune responses [85].

Understanding the genetic changes in SARS-CoV-2 also plays a critical role in assessing the effectiveness of existing vaccines and the need for booster doses. A study demonstrated that the Delta variant’s mutations could partially evade neutralizing antibodies generated by previous infection or vaccination, underscoring the importance of booster doses to maintain high levels of immunity [86]. Similarly, research on Omicron variants (BA.1, BA.2, BA.4, BA.5) shows significant sequence variability, potentially affecting their interaction with cellular receptors and immune recognition [87]. These findings underscore the importance of booster doses to maintain high levels of immunity and support the ongoing adaptation of vaccination strategies to address the evolving threat posed by new variants.

Variant verification plays a pivotal role in understanding the evolution of SARS-CoV-2. By identifying and tracking specific mutations, researchers can predict future changes and prepare for new variants, ultimately aiding in the development of effective public health strategies and interventions.

### 4.2. Role in Epidemiological Tracking

#### 4.2.1. Monitoring the Spread and Prevalence of Specific Variants

Epidemiological tracking plays a crucial role in monitoring the spread and prevalence of different SARS-CoV-2 variants within populations [88]. This process helps public health officials understand which variants are circulating and where they are most prevalent, enabling targeted interventions and efficient resource allocation. For instance, continuous genomic surveillance has been instrumental in identifying the spread of the Delta variant, which prompted increased vaccination efforts and the reintroduction of public health measures to curb transmission [30,31].

##### Monitoring the Geographic Distribution

Monitoring the geographic distribution of SARS-CoV-2 variants is essential for understanding how different variants spread across regions. As discussed earlier, studies have shown that certain variants, such as the Alpha variant (B.1.1.7), initially identified in the UK, rapidly spread to multiple countries, becoming the dominant strain in many areas [5]. Similarly, the Delta variant (B.1.617.2), first detected in India, quickly spread globally, leading to significant waves of infection in various regions [29]. Geographic tracking aided the identification of hotspots and regions at higher risk of spread, enabling targeted public health interventions such as travel restrictions (discussed below).

##### Monitoring Temporal Trends

Temporal tracking of variants involves analyzing how the prevalence of different variants changes over time [89]. This is crucial for identifying emerging variants and understanding their impact on the pandemic’s trajectory. The rapid rise of the Omicron variant (B.1.1.529) in late 2021 highlighted the need for continuous surveillance to detect and respond to new variants promptly [78]. Temporal trends also help assess the effectiveness of public health measures and vaccination campaigns over time.

##### Population-Specific Variants

Certain variants may show higher prevalence in specific populations due to factors such as travel patterns, local genetic context—especially the frequency of HLA alleles—population density, and public health measures [90]. The Beta (B.1.351) and Delta (B.1.617.2) variants were initially predominant in South Africa and India before spreading to other regions. Understanding population-specific prevalence helps tailor public health responses to the unique needs of different communities. It also aids in identifying vulnerable populations that may require additional protective measures or targeted vaccination efforts.

#### 4.2.2. Detecting Outbreaks of SARS-CoV-2 Variants

##### Early Warning Systems

Early detection of new SARS-CoV-2 variants is crucial for signaling the beginning of an outbreak. Genomic surveillance systems play a vital role in this process by continuously monitoring viral samples for new mutations [91]. For instance, the rapid identification of the Alpha variant (B.1.1.7) in the UK was facilitated by robust genomic sequencing efforts, which allowed public health officials to quickly recognize and respond to the emerging threat [92]. Early warning systems enable timely interventions, such as targeted testing, contact tracing, and isolation measures, to contain outbreaks before they become widespread.

##### Case Clustering and Hotspots

Tracking the emergence and spread of variants involves identifying clusters of cases and potential hotspots. Genomic data can reveal patterns of transmission and help pinpoint areas where a variant is spreading rapidly [93]. During the early stages of the Delta variant (B.1.617.2) outbreak in India, genomic sequencing identified specific regions with high transmission rates, prompting localized public health responses [94]. By understanding the geographic and demographic distribution of cases, health authorities can implement targeted measures to control the spread.

##### Rapid Response Strategies

Once a new variant is detected, rapid response strategies are essential to mitigate its impact. These strategies include increasing testing capacity, enhancing genomic surveillance, and deploying public health measures such as travel restrictions and quarantine protocols. The detection of the Omicron variant (B.1.1.529) in South Africa led to immediate international travel advisories and increased genomic sequencing efforts globally [78]. Rapid response strategies are informed by real-time data on variant spread and characteristics, enabling public health officials to adapt their approaches as new information becomes available.

#### 4.2.3. Evaluating Control Measures for SARS-CoV-2 Variants

##### Effectiveness of Vaccination Campaigns

Evaluating the effectiveness of vaccination campaigns is crucial in controlling the spread of SARS-CoV-2 variants. Studies have shown that vaccines significantly reduce the incidence of severe disease, hospitalization, and death, even with the emergence of new variants. For instance, it has been demonstrated that vaccination was effective in reducing the severity of illness caused by the Delta variant although breakthrough infections were more common compared to earlier strains [95,96]. Continuous monitoring of vaccine effectiveness against different variants helps update vaccination strategies, including the administration of booster doses to maintain high levels of immunity.

##### Impact of Non-Pharmaceutical Interventions (NPIs)

Non-pharmaceutical interventions (NPIs) such as lockdowns, mask mandates, and social distancing have been pivotal in controlling the spread of COVID-19. A systematic review and meta-analysis published in *The BMJ* found that mask-wearing reduced the incidence of COVID-19 by 53%, while physical distancing lowered transmission rates by 25% [97]. These interventions are particularly important when vaccine coverage is low or when new variants with higher transmissibility emerge. Thus, evaluating the impact of NPIs helps determine the most effective strategies for reducing transmission and protecting public health.

##### Adjustments to Public Health Policies

Tracking the spread of SARS-CoV-2 variants has been crucial in shaping and adjusting public health policies. For example, the rapid global spread of the Omicron variant (B.1.1.529) in late 2021 prompted many countries to reintroduce travel restrictions, implement quarantine measures, and enhance testing protocols to slow its transmission [98]. The high transmissibility of Omicron, coupled with its ability to evade immunity from prior infection or vaccination, forced policymakers to adjust their strategies swiftly. In South Korea, the government reinstated strict indoor mask mandates and limited public gatherings when Omicron subvariants began driving a surge in cases in early 2022 [99]. Similarly, Australia temporarily paused its phased reopening plans and reintroduced mandatory quarantine for international arrivals [100].

The emergence of the Delta variant (B.1.617.2) in India during early 2021 provides another example of how mutations influenced public health responses. Delta’s increased transmissibility and immune escape properties overwhelmed healthcare systems, leading to record-high case numbers and fatalities [32]. In response, many countries, including the United Kingdom and the United States, accelerated their booster dose campaigns to mitigate the variant’s impact [101,102].

In addition to large-scale measures, specific policies have been adapted based on real-time variant monitoring. For instance, the discovery of mutations like E484K and L452R, which enhance immune evasion, led to the rollout of updated testing protocols to detect these mutations more efficiently [11]. The WHO and national health agencies have also modified isolation and quarantine guidelines to reflect shorter incubation periods observed with Omicron, balancing containment efforts with societal disruptions [103].

#### 4.2.4. Global Surveillance and Collaboration in SARS-CoV-2 Variant Tracking

##### Data Sharing Platforms

Global surveillance of SARS-CoV-2 variants relies heavily on data sharing platforms like Global Initiative on Sharing All Influenza Data (GISAID). GISAID facilitates the rapid sharing of genomic data from SARS-CoV-2 samples collected worldwide [104]. This platform has been instrumental in tracking the emergence and spread of variants by providing researchers and public health officials with access to up-to-date genetic sequences and related epidemiological data [104]. The collaborative nature of GISAID allows for real-time monitoring and analysis, which is crucial for identifying new variants and understanding their potential impact on public health.

##### International Cooperation and Coordination

Effective global surveillance requires robust international cooperation and coordination. Organizations such as the WHO and the Centers for Disease Control and Prevention (CDC) work closely with national health agencies and research institutions to monitor and respond to SARS-CoV-2 variants. For instance, the WHO’s Global Influenza Surveillance and Response System (GISRS) has been adapted to include SARS-CoV-2, leveraging its existing infrastructure to enhance global genomic surveillance [105]. This coordinated effort ensures that data on new variants are rapidly shared and that public health responses are aligned across countries.

##### Standardization of Genomic Surveillance Protocols

Standardizing genomic surveillance protocols is essential for ensuring the consistency and reliability of data collected from different regions. The WHO has published guidelines and operational considerations to help countries implement effective genomic surveillance systems [106]. These guidelines cover aspects such as sample collection, sequencing methodologies, and data reporting standards. By adhering to standardized protocols, countries can contribute high-quality data to global databases, facilitating accurate tracking and comparison of variants.

#### 4.2.5. Public Health Implications of SARS-CoV-2 Variant Verification

##### Resource Allocation and Planning

Variant verification plays a critical role in resource allocation and planning for public health responses. By identifying and tracking the spread of specific variants, health authorities can allocate resources more effectively to areas most in need. For example, during the surge of the Delta variant, some regions with high transmission rates were reported to have received increased supplies of medical equipment, personnel, and vaccines [38]. However, this targeted approach is still debated, and there is a lack of consistent evidence confirming that such targeted allocations occurred uniformly across regions. While some reports suggest that specific areas were prioritized, the effectiveness and implementation of these strategies have been subject to varying interpretations and data limitations. Therefore, more detailed studies are necessary to fully assess the impact of variant-driven resource allocation. This approach, when verified, can help ensure that healthcare systems are not overwhelmed and that resources are used efficiently to manage outbreaks.

##### Policy Development and Implementation

The continuous monitoring and verification of SARS-CoV-2 variants inform the development and implementation of public health policies. Policies such as travel restrictions, quarantine protocols, and vaccination mandates can be tailored based on the prevalence and characteristics of circulating variants. For instance, the rapid spread of the Alpha variant prompted many countries to tighten travel restrictions and enhance testing requirements for incoming travelers [107]. By basing policies on up-to-date variant data, governments can implement more effective measures to control the spread of the virus.

### 4.3. Challenges and Limitations of SARS-CoV-2 Variant Verification

#### 4.3.1. Data Quality and Completeness

One of the primary challenges in variant verification is ensuring the quality and completeness of genomic data [108]. Inconsistent data collection methods, varying sequencing capacities, and differences in reporting standards across regions can lead to gaps in the data. A recently published study highlighted that incomplete or low-quality genomic data can hinder the accurate identification and tracking of variants, ultimately affecting public health responses [10]. Ensuring high-quality, comprehensive data is essential for reliable variant verification and epidemiological tracking.

#### 4.3.2. Ethical and Privacy Considerations

Genomic surveillance involves collecting and analyzing genetic information, which raises ethical and privacy concerns. Protecting the privacy of individuals whose samples are sequenced is crucial. Maintaining confidentiality and obtaining informed consent are key ethical considerations in genomic surveillance [10]. Thus, balancing the need for public health data with individual privacy rights requires careful ethical oversight and transparent policies.

#### 4.3.3. Logistical and Financial Constraints

Implementing widespread genomic surveillance and variant verification can be logistically challenging and financially demanding. The costs associated with sequencing technologies, data storage, and bioinformatics support can be substantial. Limited financial resources and logistical challenges can impede the scalability of genomic surveillance efforts [109]. Furthermore, the costs may vary widely depending on the region, with wealthier countries potentially having greater access to the necessary technologies. To ensure the sustainability and scalability of genomic surveillance, targeted investments in both funding and infrastructure are critical. This includes not only securing financial resources but also establishing the necessary technological and human resource infrastructure, ensuring equitable access to genomic surveillance capabilities, and fostering global collaboration to bridge gaps in less-resourced settings.

#### 4.3.4. Methodological Challenges in Genomic and Epidemiological Data Analysis

The analysis of genomic and epidemiological data in the context of SARS-CoV-2 is subject to several methodological limitations that can affect the accuracy and reliability of findings. One major challenge is the sampling bias as genomic surveillance often depends on samples collected from specific regions, populations, or healthcare settings. This can lead to over-representation or under-representation of certain variants, skewing the global understanding of their prevalence and characteristics [110]. Additionally, incomplete data in epidemiological studies, such as missing information on patient demographics, vaccination status, or comorbidities, limit the ability to accurately assess the impact of variants on transmissibility, severity, and vaccine efficacy [111].

Another limitation arises from lags in sequencing and reporting as delays between sample collection, genomic sequencing, and data dissemination can result in analyses that are not reflective of the current state of variant circulation. Furthermore, confounding factors, such as public health interventions, population immunity levels, and healthcare infrastructure, can influence observed epidemiological trends, making it difficult to isolate the specific effects of a variant [112]. Early analyses often rely on small sample sizes, particularly for newly emerging variants, leading to uncertainties and potential over- or underestimation of key characteristics like transmissibility or immune escape. Lastly, the rapid evolution of the virus poses an ongoing challenge as new mutations can alter variant behavior before conclusions from earlier analyses are fully integrated into public health responses [113].

To address these challenges, there is a need for enhanced genomic surveillance, improved data integration across regions, and advanced statistical models that account for confounding variables. These steps will help ensure more robust and timely insights into the behavior of SARS-CoV-2 variants and their implications for public health strategies.

## 5. Implications for Vaccine Development and Public Health

The continuous emergence of SARS-CoV-2 variants poses critical challenges for vaccine development and the implementation of effective public health strategies. Variants with mutations in key viral proteins, such as the spike protein, have demonstrated the potential to evade immune responses, increase transmissibility, and reduce the effectiveness of existing vaccines. These developments underscore the need for adaptable and forward-looking approaches to pandemic control.

### 5.1. Challenges to Vaccine Effectiveness

Mutations in SARS-CoV-2 variants, particularly those affecting the receptor-binding domain of the spike protein, can compromise the neutralizing efficacy of vaccines developed against the ancestral virus strain [114]. For instance, mutations such as E484K and N501Y, commonly observed in variants like Beta (B.1.351) and Gamma (P.1), enhance the virus’s ability to bind more tightly to the ACE2 receptor and evade neutralizing antibodies. The L452R mutation, present in Delta (B.1.617.2) and other subvariants, not only increases infectivity but also reduces vaccine efficacy by impairing antibody binding. Omicron (B.1.1.529) and its subvariants exhibit extensive mutations in the spike protein, including K417N, T478K, and G446S, which collectively reduce vaccine-induced neutralizing antibody titers, increasing the likelihood of breakthrough infections [115]. These mutations highlight the importance of mitigation strategies to sustain vaccine efficacy. Booster dose campaigns have been widely implemented to counteract waning immunity and enhance antibody titers, providing additional protection against immune-evasive variants [116]. Multivalent vaccines, designed to target multiple variants simultaneously, are also under development to broaden immune responses. Moreover, the rapid sequencing of emerging variants enables timely updates to existing vaccines, ensuring that they remain effective against newly arising strains. In addition to vaccination, non-pharmaceutical interventions and antiviral treatments continue to play a critical role in mitigating the impact of variants with immune escape capabilities. Additionally, evidence indicates that immunity wanes over time, and the ability of variants to escape immune recognition partially exacerbates this decline, necessitating widespread booster dose campaigns [117].

### 5.2. Advancing Vaccine Development

Vaccine development is evolving to address the above challenges to ensure broad and long-lasting protection against SARS-CoV-2 and its variants. Targeted vaccines designed for highly transmissible and immune-evading variants, such as Omicron-specific formulations, are being evaluated for enhanced protection [118]. Interestingly, Omicron-specific formulations—including monovalent formulations against the recombinant variant XBB 1.5 (e.g., Comirnaty)—are already available [119]. Research is also focused on developing universal coronavirus vaccines that target conserved regions of the virus, providing cross-protection against current and future variants [120]. Additionally, mRNA vaccine platforms offer a significant advantage by enabling rapid modification and deployment of updated vaccines, making them an essential tool in responding to the ongoing evolution of SARS-CoV-2 [121].

### 5.3. Vaccine Distribution and Global Health Equity

The global response to SARS-CoV-2 variants is significantly hindered by disparities in vaccine distribution, as unequal access to vaccines fosters hotspots for the emergence of new variants and perpetuates the pandemic. Addressing these inequities is crucial, with efforts focused on ensuring that low- and middle-income countries have timely access to vaccines to curb global variant evolution. Additionally, while high-income countries have implemented extensive booster dose campaigns, expanding access to booster doses on a global scale is essential for achieving long-term pandemic control and reducing the risk of variant-driven surges.

### 5.4. Long-Term Pandemic Preparedness

The SARS-CoV-2 pandemic underscores the critical need for sustained global investment in pandemic preparedness to strengthen resilience against future threats. Establishing robust frameworks for future pandemics, including strategies such as vaccine stockpiling, enhancing manufacturing capabilities, and developing rapid response systems, is pivotal for effective containment and mitigation efforts. Furthermore, global collaboration remains indispensable as continued international cooperation enables the sharing of data, resources, and expertise, ensuring a coordinated and comprehensive response to the emergence of evolving variants.

The emergence of SARS-CoV-2 variants demands a multifaceted approach that integrates advances in vaccine development, equitable distribution, and adaptive public health policies. By addressing current challenges and strengthening pandemic preparedness, the global community can mitigate the impact of future variants and build a more resilient health infrastructure.

## 6. Lessons from the Pandemic: Toward a Unified Global Response

The end of the pandemic, as declared by the WHO, provides an opportunity to evaluate the global response and draw lessons to prepare for future pandemics. One of the most striking revelations was the disparity in resources and capabilities among countries, which significantly influenced their ability to monitor, respond to, and mitigate the effects of the disease. Wealthier nations often implemented advanced surveillance measures, such as systematic genotyping, to track viral mutations and assess disease progression. In contrast, resource-limited countries lacked the economic means to deploy similar strategies, creating blind spots in the global understanding of the pandemic’s evolution. This uneven monitoring capability delayed coordinated international responses and allowed the virus to spread unchecked in certain regions. To address this, international funding mechanisms and shared genomic databases must be developed to provide equitable access to surveillance technologies and expertise.

Another critical lesson is the need for an inclusive global surveillance network. Fragmented systems across nations revealed vulnerabilities in detecting and responding to emerging pathogens. Countries with robust healthcare infrastructure could maintain effective monitoring, while those with limited resources struggled, often at the cost of higher transmission rates and prolonged outbreaks. A globally integrated disease surveillance system, coordinated by entities like the WHO, is essential to standardize data collection and ensure real-time information sharing. Support for low-income nations through technical assistance, funding, and capacity building will strengthen early warning systems and enhance global preparedness.

Disparities in access to vaccines, treatments, and protective equipment further underscored the inequities during the pandemic. Wealthier nations secured resources rapidly, leaving economically disadvantaged countries with delayed access to life-saving interventions. This inequity not only prolonged outbreaks in these regions but also increased the risk of new variants emerging, threatening global health security. Moving forward, global agreements such as a pandemic treaty should prioritize equitable access to essential resources. Mechanisms for stockpiling and fair distribution of vaccines, diagnostics, and medications must be established to ensure that no country is left behind in future pandemics.

The pandemic also highlighted the importance of strengthening healthcare systems, particularly in resource-limited settings. Weak healthcare infrastructures struggled to manage surges in cases, resulting in higher mortality rates and severe socio-economic consequences. Investments in healthcare capacity, workforce training, and public health education are critical to enhancing resilience. Partnerships between high- and low-income countries can provide technical and logistical support to build stronger systems that are better prepared for future crises.

Geopolitical tensions and fragmented responses during the pandemic hindered effective global coordination, delaying collective action. Multilateral organizations like the WHO must be empowered to foster trust between nations and coordinate unified responses. Clear frameworks for information sharing, equitable decision-making, and action plans should be developed to avoid delays and ensure global collaboration. Additionally, leveraging advances in technology, such as artificial intelligence, mobile health tools, and real-time data analytics, is vital for efficient pandemic management. However, these technologies must be accessible to all countries, necessitating initiatives to democratize innovation and integrate technological solutions into global public health systems.

Ultimately, the pandemic has demonstrated that infectious diseases recognize no borders. A unified global strategy is required to address disparities, foster collaboration, and enhance preparedness. By investing in equitable resource allocation, inclusive surveillance systems, and coordinated multilateral efforts, the global community can build a resilient and effective response to future pandemics.

## 7. Conclusions

The ongoing evolution of SARS-CoV-2, driven by the emergence of variants such as Alpha, Beta, Gamma, Delta, and Omicron, underscores the virus’s adaptability and its significant implications for public health. Genetic mutations, particularly in the spike protein, have enhanced the virus’s transmissibility, immune evasion, and virulence, challenging global healthcare systems and vaccine effectiveness.

Epidemiological tracking and variant verification remain critical for monitoring the spread, understanding the impact of these variants, and informing public health interventions. Genomic surveillance has proven invaluable in identifying variants and shaping strategies to mitigate their effects, from adapting vaccine formulations to implementing targeted containment measures. However, challenges such as data quality, resource limitations, and vaccine inequity persist, highlighting the need for global collaboration and investment in robust pandemic preparedness.

Looking forward, advancements in universal vaccines and adaptive technologies like mRNA platforms offer hope in addressing current and future variants. By prioritizing equitable access to vaccines and fostering international cooperation, the global community can effectively combat the pandemic and strengthen resilience against emerging infectious diseases.

To address challenges in resource-limited contexts, vaccine design should prioritize broad neutralization by targeting conserved regions of the SARS-CoV-2 spike protein, ensuring efficacy against multiple variants. Heat-stable and low-cost vaccine platforms, such as protein subunit or viral vector vaccines, can improve accessibility, while multivalent and dose-sparing strategies can expand coverage. For surveillance, decentralizing genomic sequencing to regional centers and leveraging mobile labs can enhance variant tracking. Integrating syndromic surveillance with portable diagnostic tools and fostering international collaboration for data sharing can further strengthen monitoring. Optimizing real-time data systems and focusing on sentinel surveillance in strategic locations can ensure efficient and cost-effective variant detection and response.

## Figures and Tables

**Table 1:** Characteristics of SARS-CoV-2 variants of concern (VOCs).

Variant	WHO Label	Pango Lineage	Date of Emergence	Key Mutations (Spike Protein)	Transmissibility	Pathogenicity	Immune Escape Potential	Impact on Vaccine Efficacy
**Alpha**	B.1.1.7	United Kingdom	September 2020	N501Y, D614G, 69-70del, P681H	~50% more transmissible than the original strain	Slightly increased severity compared to earlier strains	Moderate immune escape (some reduction in neutralization)	Minor reduction in efficacy; vaccines remain effective for severe disease
**Beta**	B.1.351	South Africa	May 2020	K417N, E484K, N501Y, D614G	~50% more transmissible than the original strain	No significant increase in severity observed	High immune escape, especially due to E484K	Significant reduction in neutralization by some vaccines; boosters recommended for enhanced protection
**Gamma**	P.1	Brazil	November 2020	K417T, E484K, N501Y, D614G	Increased transmissibility (data not precise)	No significant increase in severity	Moderate immune escape due to E484K	Moderate reduction in efficacy; boosters can improve protection
**Delta**	B.1.617.2	India	October 2020	L452R, T478K, D614G, P681R	Highly increased (up to 2x more than Alpha)	Possible increase in severity, particularly unvaccinated	Moderate immune escape	Reduced efficacy, especially after one dose; full vaccination and boosters recommended
**Omicron**	B.1.1.529	South Africa	November 2021	S371L, S373P, S375F, T376A, G496S, Q498R, N679K, P681H, D796Y	Extremely high transmissibility; surpasses Delta	Lower severity on average, though risks remain for unvaccinated and high-risk groups	High immune escape due to multiple mutations in RBD (e.g., E484A, Q493K)	Significant reduction in vaccine efficacy for infection; boosters enhance protection against severe disease
